# Immune-related RNA signature predicts outcome of PD-1 inhibitor-combined GEMCIS therapy in advanced intrahepatic cholangiocarcinoma

**DOI:** 10.3389/fimmu.2022.943066

**Published:** 2022-09-09

**Authors:** Tian-mei Zeng, Yu-fei Pan, Zhen-gang Yuan, Dong-sheng Chen, Yun-jie Song, Yong Gao

**Affiliations:** ^1^ School of Medicine, Tongji University, Shanghai, China; ^2^ Department of Oncology, Eastern Hepatobiliary Surgery Hospital, Shanghai, China; ^3^ International Cooperation Laboratory on Signal Transduction, Eastern Hepatobiliary Surgery Hospital, Shanghai, China; ^4^ Jiangsu Simcere Diagnostics Co., Ltd, The State Key Laboratory of Translational Medicine and Innovative Drug Development, Nanjing, China; ^5^ Department of Oncology, Shanghai East Hospital, Shanghai, China

**Keywords:** intrahepatic cholangiocarcinoma, immunochemotherapy, immune-related signature, tumor microenvironment, prognosis

## Abstract

**Background:**

Immune checkpoint inhibitor (ICI)-combined chemotherapy in advanced intrahepatic cholangiocarcinoma has been proved to have more efficacy in a series of clinical trials. However, whether the tumor microenvironment (TME) plays a vital role in immune-combined therapy has not been rigorously evaluated.

**Methods:**

Firstly, we assayed the immunogenic properties of GEM-based chemotherapy. Then, 12 ICC patients treated with PD-1 inhibitor (sintilimab) combined with gemcitabine and cisplatin (GemCis) from a phase 2 clinical trial (ChiCTR2000036652) were included and their immune-related gene expression profiles were analyzed using RNA from baseline tumor samples. Immune-related signature correlating with clinical outcome was identified according to the 12 ICC patients, and its predictive value was validated in an ICC cohort with 26 patients. Multiplexed immunofluorescence (mIF) and flow cytometry (FCM) analysis were performed to evaluate the immune-related molecules with therapeutic outcomes.

**Results:**

GEM-based chemotherapy induced immunogenic cell death of cholangiocarcinoma cells, together with increased CD274 expression. In an ICC cohort, we found that upregulation of immune-checkpoint molecules and immune response-related pathways were significantly related to better clinical outcome. On the contrary, baseline immune-cell proportions in tumor tissues did not show any correlation with clinical benefit between responders and non-responders. Immune-related signature (including six genes) correlating with clinical outcome was identified according to the 12 ICC patients, and its predictive value was validated in a small ICC cohort with 26 patients.

**Conclusion:**

Immune-related RNA signature predicts the outcome of PD-1 inhibitor-combined GEMCIS therapy in advanced intrahepatic cholangiocarcinoma, which could be tested as a biomarker for immune-chemotherapy in the future.

## Introduction

Intrahepatic cholangiocarcinoma (ICC) is a highly aggressive malignancy arising from secondary biliary epithelial cells, featuring different prognoses and genetic landscapes compared with other bile tract cancers (BTCs) (1). Surgical resection is the only potential curative way but not suitable for patients with advanced stages. Palliative chemotherapy such as gemcitabine combined with cisplatin (GemCis) (2) and other gemcitabine-based regimens (3, 4) have been recommended as first-line treatments for advanced ICC patients and acquired an objective response rate (ORR) up to 30%. However, the second-line setting remains varied currently. Patients with *FGFR2* infusion could benefit from *FGFR2* inhibitors and achieved an ORR over 30%, but only 3%~15% of the patients bear this mutation (5). Alternative therapies including other chemo or target therapies could not bring long-term survival benefit for ICC patients (6, 7).

Cancer immunotherapy, especially immune checkpoint inhibitors (ICIs), has emerged as the most promising treatment for many malignant diseases. Prior studies with convincing evidence have shown that ICI alone could not benefit BTC patients (8). Several trials with a small sample size had a promising ORR of 13% to 22% in response to ICIs (9, 10), which dropped to 5.8% as the number of patients increased to 104 (10). Recently, programmed death-ligand 1 (PD-L1) inhibitor (durvalumab) in combination with GemCis in advanced BTC patients indicated an improving response rate of about 10% to 26.7% compared to GemCis in a phase 3 randomized clinical trial (11). While immune-chemo combination therapy has been evaluated globally in BTC patients, the determinant that is relevant to a favorable outcome of this combination treatment is still undefined. PD-L1 expression, DNA damage repair (DDR) pathway-related gene mutation, and tumor microenvironment (TME) have all been reported as predictors in different studies but only offer limited insight (12–14).

Herein, we firstly verified that gemcitabine treatment could induce PD-L1 expression on cholangiocarcinoma cell lines *in vitro*. Then, we specifically examined gene expression in the TME, using RNA isolated from baseline tumor tissue samples which were obtained from ICC patients who received programmed cell death-1 (PD-1) inhibitor combined with GemCis as first-line therapy. Signature related to T-cell migration, response to biotic stimulus, and regulation of innate immune response were initially delineated in a small ICC cohort with 12 patients then confirmed and refined in a larger independent cohort. Our data probably indicate that a tumor microenviroment characterized by active T-cell migration and response to biotic stimulus or innate immunity is a common feature of the environment that has better response to immune-chemo combined therapy in ICC patients.

## Materials and methods

### Patients

Twelve patients were enrolled from the ChiCTR2000036652 trial approved by the Research Ethics Committee of Eastern Hepatobiliary Surgery Hospital. All patients were diagnosed as advanced ICC, and the possibility of microsatellite instability high (MSI-H) was excluded. All participants gave PD-1 antibody (sintilimab, 200 mg/21 days) combined with GemCis (gemcitabine: 1,000 mg/m^2^ days 1, 8; and cisplatin: 25 mg/m^2^ days 1, 8) as their first-line regimen after screening for contraindication. Tumor tissues were acquired by liver biopsy before treatment. Written informed consent was obtained from all patients.

### Cell culture

HuCCT1 and TFK1 cell lines were kindly provided by S.-Q. Zou, Tongji Hospital, Huazhong University of Science and Technology. The cells were cultured in RPMI 1640 medium supplemented with 10% FBS, penicillin (100 IU/ml) (Gibco), and streptomycin (100 μg/ml) (Gibco) and were maintained at 37°C and 5% CO_2_. To generate GEM-resistant TFK1 cells, the cells were treated with GEM (Selleck, 5 μM) for 1 week, and the live cells were washed and retreated with GEM (10 μM) for another week. The rest of the cells were collected and reseeded into a 96-well plate in the presence of GEM (10 μM) to generate monoclonal antibodies. The cell viability with the indicated drugs was measured by CellTiter-Glo^®^ Luminescent Cell Viability Assay (Promega) according to the manufacturer’s instructions.

### Sample preparation

Tumor tissues and matched blood specimens were sequenced at a CAP-certified genomics laboratory in China (Simceredx, Nanjing, China). For FFPE samples, only those samples harboring above 20% of tumor cell content were considered qualified, and subsequent genomic profiling was performed.

### DNA extraction and library preparation

Three commercial kits were used for the DNA extraction. Genomic DNA (gDNA) of formalin-fixed and paraffin-embedded (FFPE) tissues and fresh tissues was extracted using the Tissue Sample DNA Extraction Kit (Kai Shuo). Genomic DNA of leukocyte was extracted using MagMAX™ DNA Multi-Sample Ultra Kit (Thermo). Cell-free DNA (cfDNA) of plasma was extracted using MagMAX™ Cell Free DNA Isolation Kit (Thermo). All of the extraction procedures were performed following the manufacturer’s instructions. DNA was quantified on a Qubit fluorometer with Qubit dsDNA HS Assay Kit (Thermo), and its quality was evaluated by Agilent 4200 TapeStation (Agilent).

The probe hybridization capture method was used for library construction. Commercial reagents and customized probe were used for library construction and hybridization capture. In brief, 15–200 ng gDNA was sheared into 200~350 bp by fragmentation enzymes. Indexed paired-end adaptors for the Illumina platform were self-developed and customized (Simceredx). End repair, A-tailing, and adaptor ligation of sheared DNA and cfDNA were respectively performed using KAPA HyperPlus DNA Library Prep Kit (Roche Diagnostics) and VAHTS™ Universal DNA Library Prep Kit for Illumina^®^ (Vazyme). Unligated adaptors were removed by the size selection function of Agencourt AMPure XP beads (Beckman Coulter). The ligation products were PCR amplified to form a pre-library for hybridization. The final library was quantified on Qubit Fluorometer with Qubit dsDNA HS Assay Kit (Thermo Fisher), and its quality was evaluated by Agilent 4200 TapeStation (Agilent).

### Library sequencing and bioinformatics analysis

The qualified DNA libraries were sequenced on an Illumina NovaSeq 6000 platform (Illumina, San Diego, CA), and 150-bp paired end reads were generated. Base calls from Illumina NovaSeq 6000 were conducted to FASTQ files. The software fastp (v.2.20.0) was used for adapter trimming and filtering of low-quality bases (15). The BWA-MEM (v.0.7.17) algorithm was performed to align to the reference genome (UCSC’s hg19 GRCh37) (16). Duplicate reads from PCR were excluded using Dedup with Error Correct. SNVs/InDels were called and annotated *via* VarDict (v.1.5.7) (17) and InterVar (18), then the variants were filtered against the common SNPs in the public database including 1000 Genome Project (Aug 2015) and Exome Aggregation Consortium (ExAC) Browser28 (v.0.3). CNVs and fusions were analyzed by CNVkit (dx1.1) (19) and factera (v1.4.4) (20), respectively.

### TMB calculation

Non-synonymous somatic mutations, including missense, nonsense, splice-site, inframe, and frameshift mutations, which may be functional, were included in our analyses. TMB was calculated as the number of non-synonymous somatic mutations.

### Transcriptional profiling and analysis of FFPE samples

The NanoString nCounter Analysis System belongs to the third generation of gene expression detection technology, using a new molecular barcoding technology. The experiment was performed directly to digitally display the amount of gene expression in the sample with high sensitivity and accuracy. Briefly, two kinds of probes (capture probe and report probe) were used to specifically capture the target gene and then form a probe/target molecular fluorescence complex. Each complex represented different mRNA molecules. FFPE samples within 1 year were collected for testing. The proportion of tumor cells should be more than 30%. Total cellular RNA was extracted from FFPE samples using the QIAGEN FFPE RNeasy Kit (QIAGEN GmbH, Hilden, Germany) and was quantified using the NanoDrop ND1000 spectrophotometer (Thermo Fisher Scientific). A customized code set consisting of a 289-gene panel related to tumor, immune regulation, and tumor microenvironment was used. Total (100 ng) cellular RNA was hybridized to the NanoString customized code set at 65°C overnight (16 h). Thereafter, the mixture was loaded onto the nCounter Prep Station for subsequent processing, then gene expression data were generated using the nCounter™ Digital Analyzer. The housekeeping genes were employed to normalize the expression values, as recommended by the manufacturer, using nSolver 2.6 software. The expression levels of 289 immune-related genes, including housekeeping genes, are listed in **Supplemental Table 1**

### RNA extraction and bulk RNA-seq of cultured cell lines

Total RNAs of GEM-resistant were extracted using RNeasy Micro Kit (Cat# 74004, Qiagen) following the manufacturer’s instructions and checked for an RIN number to inspect RNA integrity using an Agilent 2100 Bioanalyzer (Agilent Technologies, Santa Clara, CA, US). Qualified total RNA was further purified using RNAClean XP Kit (Cat A63987, Beckman Coulter, Inc., Kraemer Boulevard Brea, CA, USA) and RNase-Free DNase Set (Cat#79254, QIAGEN, GmbH, Germany). The RNA sequencing libraries were prepared using the NEBNext Ultra II RNA Library Prep Kit for Illumina using the manufacturer’s instructions (New England Biolabs). Final libraries were sequenced on a NovaSeq 6000 with 2 × 150 bp paired-end sequencing. For each sample, RNA-seq clean reads were obtained that were mapped using HISAT2 (hierarchical indexing for spliced alignment of transcripts) v2.0.477. Sequencing read counts were calculated using StringTie (v.1.3.0). Then, expression levels from different samples were normalized by the Trimmed Mean of M-values (TMM) method. The normalized expression levels of different samples were converted to FPKM (Fragments Per Kilobase of transcript per Million mapped fragments). The edgeR package of R was used to analyze the difference between intergroup gene expressions, the P-values were calculated, and the multiple-hypothesis test was performed. The P-value threshold was determined by controlling the FDR (false discovery rate) with the Benjamini algorithm. Genes with false discovery rate <0.05 and absolute value of fold change ≥1.5 were selected as differential. The volcano plot for the DEGs was visualized by the ggplot2 package (3.3.3).

### Gene set enrichment analysis and immune cell type analysis

Gene set enrichment analysis (GSEA) was performed using GSEA v.4.2.3 (https://www.gsea-msigdb.org/gsea/) with default parameters. The Hallmark gene set, GOBP gene set, and WP gene set were collected from the Molecular Signatures Database (MSigDB) collection. FDR q-values <0.1 were used to identify significantly enriched pathways. Marker genes of macrophages, exhausted CD8 T cells, T cells, CD8 T cells, neutrophils, mast cells, cytotoxic cells, Tregs, NK CD56dim cells, NK cells, CD45, and Th1 cells were retrieved from the method previously reported (21–23). All immune cell type scores were calculated as the arithmetic mean of the constituent genes, and the difference of these scores between the responders and non-responders was examined using the Wilcoxon test, with a *p* value less than 0.05 which was considered to be statistically significant.

### Multiplexed immunofluorescence

We used the Leica BOND RX for multiplex immunofluorescence staining, according to the manufacturer’s instructions. Sections were washed in PBS, blocked with 10% normal goat serum, permeabilized in PBS containing 0.1% Triton X-100 (PBT) for 2 h, and incubated in primary antibody diluted in blocking solution overnight at 4°C. Sections were subsequently washed in PBT and incubated in secondary antibody for 2 h at 37°C. Finally, coverslips were applied. The primary antibodies used for immunostaining were anti-CD4 (Ventana, SP35) and anti-CD8 (Ventana, SP57). PD-L1 expression was determined using a Ventana PD-L1 IHC pharmDx Kit (SP263). The PD-L1 expression level was evaluated by tumor proportion score (TPS). DAPI (Sigma) was used to stain the nuclei.

### Flow cytometry

Human cholangiocarcinoma cell lines HuCCT1 and TFK1 were treated with gemcitabine (10 μM) or oxaliplatin (3 mg/ml) for the indicated time; the cells were detached and stained with anti-PDL1-APC (BioLegend, 323124). For peripheral lymphocyte assay, human blood cells were collected and mononuclear cells were purified with Ficoll gradient centrifugation; the cells were stained with anti-CD4-APC, anti-CD8a-FITC, anti-CD3-PerCPCy5.5, anti-CD19-PE, anti-CD56-PECy7, and anti-CD28-PB (BioLegend). Flow cytometry assay was performed on BD LSRFortessa and analyzed with FlowJo (BD).

### Detection of immunogenic cell death

The analysis of cell surface CRT was performed by flow cytometry. The cells were collected and incubated with primary mouse anti-CRT (Abcam, ab22683) for 30 min at 4°C. Then, cells were washed and stained with the secondary antibody. The live cells were gated as DAPI^-^. HMGB1 in culture supernatants was measured by ELISA (Solarbio Life Science, China). ATP levels in culture supernatants were measured by CellTiter-Glo^®^ Luminescent Cell Viability Assay.

### Real-time PCR

Total RNA from tumor cells was extracted with TRIzol and reverse transcribed using M-MLV Reverse Transcriptase (Invitrogen). The cDNAs were analyzed by real-time quantitative PCR (RT-qPCR) using SYBR Green (Roche) according to the manufacturer’s instructions using the LightCycler 480 Real-Time PCR system (Roche). Expression was normalized to the expression of ACTB.

### Statistics

Continuous data with a normal distribution were presented as means and standard deviations and that with a screwed distribution were presented as medians and ranges. Categorical data were presented as frequencies or percentages. Survival curves were drawn using the Kaplan–Meier method. Progression-free survival (PFS) time was defined as the time between the diagnosis and confirmed disease progression of the patient. Overall survival time was defined as the time between the diagnosis and death of the patient. In this study, “responders” was defined as patients with any tumor regression from the baseline and “non-responders” was defined as patients with any tumor size increase from the baseline. Calculation of the area under the receiver operating characteristic (ROC) curve was used as a measure of discriminatory ability for the signature scores. A *p*-value < 0.05 was considered statistically significant. All statistical analyses were performed using GraphPad (V 8.0), R (V. 4.1.0), and R Bioconductor packages (https://www.r-project.org).

## Results

### Gemcitabine-based chemotherapy-induced immunogenic cell death

**Figure 1:**
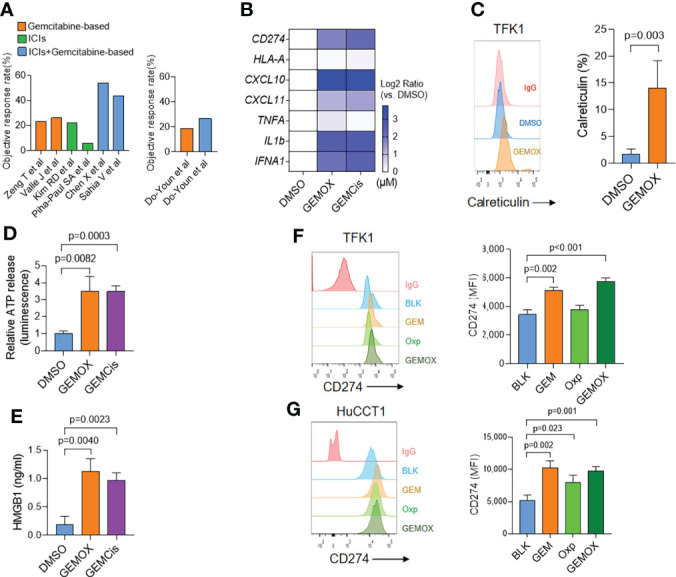
Gemcitabine-based treatment induces immunogenic cell death in cholangiocarcinoma. **(A)** Retrospectively analyzed patients received gemcitabine (GEM)-based regimens with or without ICI inhibitors. **(B)** Real-time PCR assay of TFK1 cells treated with DMSO, GEM (10 μM) + oxaliplatin (5 μM), or GEM (10 μM) + cisplatin (5 μM) for 48(h) **(C)** The cell surface calreticulin expression of cells treated as in real-time PCR results. **(D)** The supernatant ATP levels of cells treated as in real-time PCR results. **(E)** The supernatant HMGB1 levels of cells treated as in real-time PCR results. **(F)** The FCM and quantification of CD274 expression in TFK1 cells treated with GEM (10 μM), oxaliplatin (5 μM), or combined for 24(h) **(G)** The FCM and quantification of CD274 expression in HuCCT1 cells treated with GEM (10 μM), oxaliplatin (5 μM), or combined for 24 h.

We first retrospectively analyzed patients who received gemcitabine (GEM)-based regimens with or without ICI inhibitors from the public clinical trials and in our department. ICI or chemotherapy treatment showed the objective response rate (ORR) ranged from 5.8% to 26.1% (2, 9, 10, 24). However, the combination therapy showed the ORR to be 43.8%–54% (25, 26) (**Figure 1A**), suggesting that GEM-based chemotherapy could improve the ICI treatment. These results were further confirmed by the clinical trial performed by Do-Youn et al. (11) (**Figure 1A**).

We then tested the immunogenic properties of GEM-based chemotherapy. The TFK1 cells were treated with GEM (10 μM) plus oxaliplatin (5 μM) or cisplatin (5 μM) for 48 h. These treatments induced around 50% of cell death. Real-time PCR assay demonstrated that both GEM-based chemotherapy induced type I IFN (*IFNA1*) and chemokine (*CXCL10*, *CXCL11*) expression (**Figure 1B**). Meanwhile, GEM-based chemotherapy elevated cell surface calreticulin (CRT) expression (**Figure 1C**) and cellular ATP and HMGB1 release (**Figures 1D, E**). Together, these results suggest that GEM-based chemotherapy could induce immunogenic cell death in cholangiocarcinoma cells. Interestingly, we found that GEM-based chemotherapy induced tumoral PD-L1 expression at both transcriptional (**Figure 1B**) and protein levels (**Figures 1F, G**). We also found an increasing expression of PD-L1 in GEM-resistant TFK1 cells, together with enrichment of inflammatory response (**Supplement Figure 1A–F**).

According to the above results, we speculate that GEM-based chemotherapy could induce the immunogenic cell death of tumor cells and higher expression of PD-L1, which might help to boost the effect of ICIs.

### Characteristics of the 12 ICC patients receiving anti-PD-1 plus GemCis treatment

The clinical characteristics of patients in our cohort are shown in **Table 1**. The median age was 60 years, and most of them were in advanced stages with hematogenous metastasis to extrahepatic organs. All patients were in good performance status, and the infective rate of hepatitis B virus was low.

**Table 1:** Patients Baseline Characteristics.

Patients characteristics	All patients (n=12)
Age, median (years)	57.5 (48-74)
Sex, male, (n%)	9 (75)
Disease stage, (n%)	
Locally advanced	1 (8.3)
Metastastic	11 (91.7)
ECOG PS*	
0	8 (66.7)
1	4 (33.3)
CA199≥ upper limit of normal, n (%)	10 (83.3)
Hepatitis B, n (%)	3 (25)

*Eastern Cooperative Oncology Group Performance Status.

**Figure 2:**
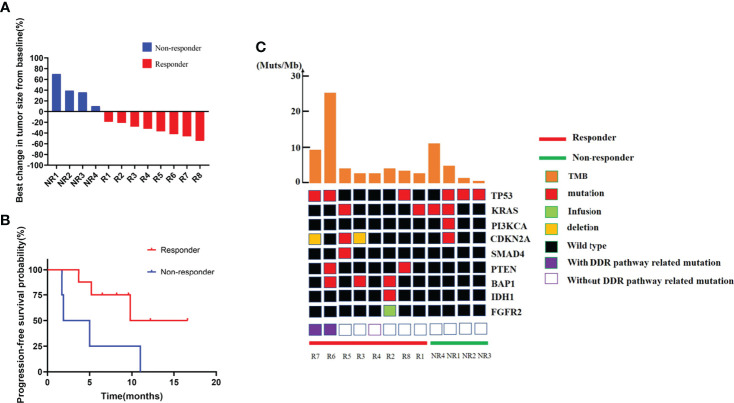
Efficacy, PFS, and genomic information of the ICC cohort. **(A)** Best change in target lesion size from baseline. **(B)** PFS among patients with different response. **(C)** Genetic characteristics and total tumor burden comparation between responders and non-responders.

Patients were divided into two groups as responders and non-responders. Response was defined as any level of tumor regression from baseline (**Figure 2A**). In this cohort, four patients got a continuous disease progression during treatment while the other eight acquired different levels of tumor regression. PFS also showed a significant difference between two groups (**Figure 2B**), which was 13.2 months in responders and 3.45 months in non-responders (*p* = 0.0448, HR: 0.25, 95% CI: 0.04 to 1.38).

### Genomic alteration and tumor mutation burden comparison between responders and non-responders

DNA sequence from the pretreated ICC tumor biopsies revealed a similar mutation landscape as reported in another BTC cohort (27) (**Figure 2C**). *TP53* and *KRAS* mutations were still the most common alterations (28, 29), and their distributions were comparable in two groups. *IDH1* mutation and *FGFR2* fusion, the two famous therapeutic targets in ICC (30, 31), were detected in patients. Other high frequently mutated gene alterations including *PTEN*, *PI3KCA*, *BAP1*, *CDKN2A*, and *SMAD4* were also found. However, due to the small number of patients enrolled, single-gene alternations did not show any correlation with immune-chemotherapy benefit.

The median TMB of the baseline tumors was 3.195 Muts/Mb, just a little bit higher than in a previous report of a large Chinese cohort (32). We still did not find a difference in TMB between the two groups. However, two patients without MSI-H or *POLE* mutation represent remarkably higher TMB, which were 25.74 and 11.35 Muts/Mb, respectively (**Figure 2C**). It is very interesting that these two patients displayed completely different responses to combined therapy (one with 42% shrinkage and another with a 70% increase of tumor size), resulting in various clinical outcomes.

### PD-L1 expression in responders and non-responders

**Figure 3:**
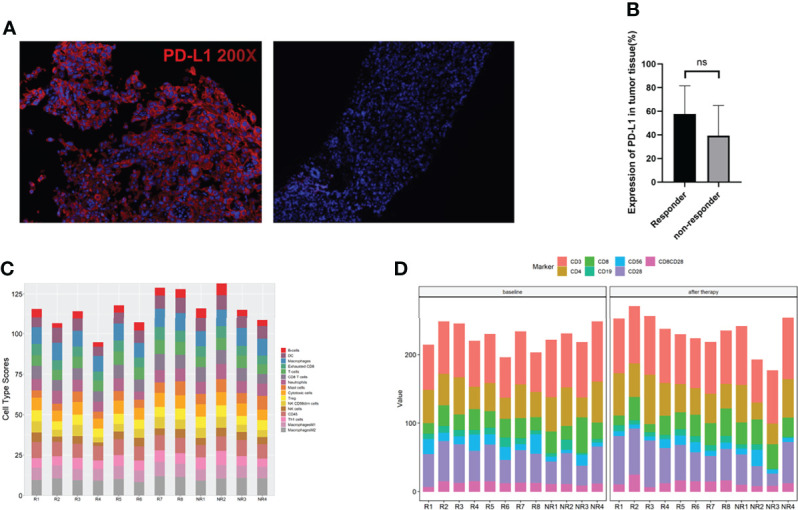
Surface biomarkers of CD4, CD8, and PD-L1 by multiplexed immunohistochemistry (mIHC) and immune cell analysis. **(A)** Typical micrographs of surface biomarkers, at ×200 magnification. PD-L1 (red). Left: PD-L1-positive tumor; right: PD-L1-negative tumor. **(B)** Comparation of PD-L1 expression between responders and non-responders. **(C)** Immune cell type score in responders and non-responders. **(D)** Immune cells in peripheral blood at baseline and 2 cycles after therapy in responders and non-responders. ns, no significance.

PD-L1 expression in the pretreated tumor samples of this cohort represented a surprising higher proportion than previous report through a more sensitive measurement—multiplexed immunofluorescence (mIF) (**Figure 3A**). Ten out of twelve patients were PD-L1-positive (staining rate more than 30%). A super high expression—over 90%–was detected in two patients who both acquired and maintained partial response (PR) for more than 10 months. However, a significant association between PD-L1 expression and response to immune-combined therapy was not confirmed in this small sample group (*p* = 0.2588) (**Figure 3B**).

### Immune cell type score and peripheral blood lymphocyte subsets in responders and non-responders

The abundance of immune cells at baseline were assessed by RNA NanoString with a 289-gene panel and 56 immune cell type-specific genes (gene list details are shown in **Supplemental Table 2**). Interestingly, we found that all the immune cell types assayed did not have a significant difference between responders and non-responders, including M1/M2 macrophages, Tregs, cytotoxic cells, and exhausted CD8 cells (**Figure 3C**). We then assayed the peripheral lymphocyte changes between patients pretreated and those receiving 2 cycles of combined therapies by flow cytometry. The results are shown in **Figure 3D**. No differences of the immune cell types tested at baseline and after therapy were found between responders and non-responders.

### Immune-related molecules and pathways in responders

**Figure 4:**
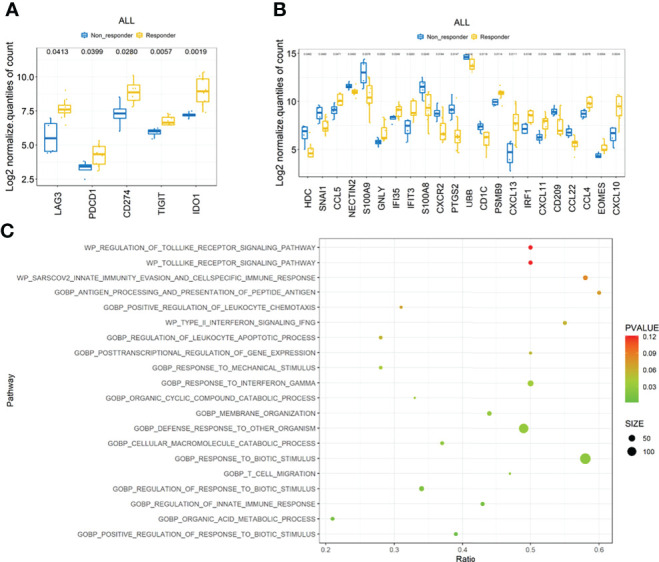
Transcription features the ICC cohort. **(A)** Comparison of checkpoint molecules including PD-1, PD-L1, LAG3, TIGHT, and IDO1 between responders and non-responders. **(B)** Comparison of other immune-related molecules that were significantly different between responders and non-responders. **(C)** Top 20 immune-related pathways enriched by gene set enrichment analysis (GSEA).

We then compared the gene expression profiles between responders and non-responders according to the RNA NanoString data. The immune checkpoint molecules, including *LAG3*, *PD1(PDCD1)*, *PDL1(CD274)*, and *TIGIT*, were significantly upregulated in responders, together with the immune inhibitory molecule *IDO1* (**Figure 4A**). Other genes related to antigen presentation and cell migration were found significantly different in responders and non-responders as shown in **Figure 4B**. Gene set enrichment analysis (GSEA) indicated that several crucial immune-associated signalings were significantly enriched in responders, such as response to interferon gamma (**Figure 4C**). The genes in enriched pathways are listed in **Supplemental Table 3**.

### Genetic signature predicts immunocombined therapy outcomes

**Figure 5:**
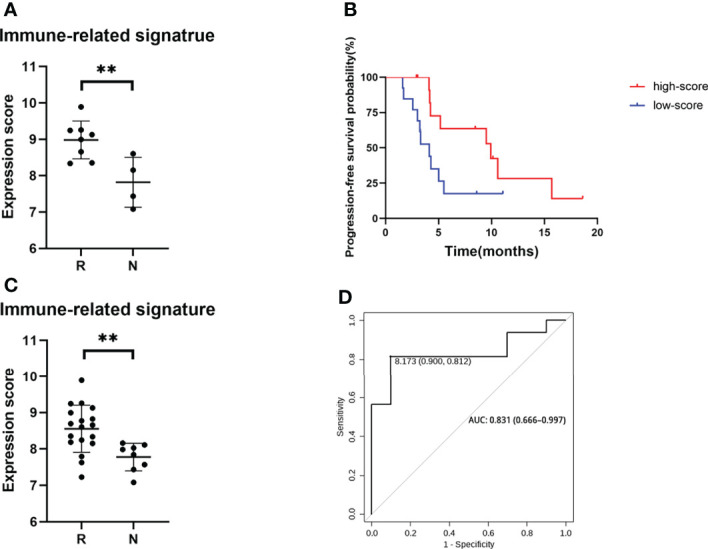
Genomic signature of the ICC cohort. **(A)** Expression score of six core genes between responders and non-responders in 12 selected patients from clinical trial. **(B)** PFS of patients with high score and low score of six core genes. **(C)** Expression score of six core genes validated in a cohort of 26 ICC patients. **(D)** ROC curves of sensitivity and specificity for the cohort of 26 ICC patients. **p < 0.01.

The top three enriched pathways including response to biotic stimulus (*p* = 0.002), regulation of innate immune response (*p* = 0.009), and T-cell migration (*p* = 0.022) were initially selected to delineate immune-related signature. The core genes (*PSMB10*, *PSMB9*, *LAG3*, *CCL5*, *IFI35*, and *SH2D1A*) enriched at least in two of these three pathways were chosen to generate a six-gene set, referred to as “immune-related signature”, to separate the responders and non-responders in the pilot cohort of 12 patients (**Figure 5A**, *p* = 0.008). The signature score was calculated based on the mean expression values (at the log2 scale) of the selected core genes. To evaluate the predictive value of these six-gene signature score in the clinical outcome of ICC patients receiving immunochemical combined therapy, we enrolled another 26 ICC patients with the same regimen. The six-gene expression profiles in the pretreated tumor tissues were performed. Progression-free survival analysis of the 26 patients based on the six-gene set score showed a significant difference (9.9 *vs*. 4.1 months, *p* = 0.0379) and a higher score associated with better prognosis (**Figure 5B**). Higher levels of the six-gene score were found in responders than in non-responders in this validation cohort (*p* = 0.0044) (**Figure 5C**). Furthermore, ROCs for response status over the range of the signature scores demonstrated good discriminatory ability of the signatures (**Figure 5D**). Areas under the ROC curves and their 95% CIs were 0.831 (0.666–0.997) for ICC patients. These results demonstrated that the gene expression pattern in pretreatment tumor biopsy specimens could predict posttreatment clinical outcomes to immunochemical therapy. Moreover, these results suggest a potential for a high discriminatory value of immune-related six-gene signatures.

## Discussion

GemCis is the only standard treatment for ICC patients. The ORR of gemcitabine-based chemotherapies in our retrospective cohort was 23%, similar to the ABC-06 study (2, 24). BTC patients treated by single ICI displayed dissatisfied ORR ranging from 5.8% to 22% (9, 10). However, recent research of immune-combined therapy in BTC showed an increasing ORR amount to 43.8%~54% (25, 26). Whether and how chemotherapy facilitate the anti-PD1 therapy needs exploration. In our study, we have proved that gemcitabine–based chemotherapy could induce immunogenic cell death and PD-L1 expression on ICC cell lines. These results provide a possible mechanism of improved effect when immunotherapy was combined with GemCis (11).

In our research, 12 ICC patients from a phase 2 clinical trial (ChiCTR2000036652), aiming to evaluate the efficacy and safety of sintilimab (a kind of anti-PD-1 antibody) plus GemCis as first-line treatment in advanced BTC patients, were enrolled as the exploratory cohort. Patients were divided into two groups due to their response to immune-combined therapy, as shown in **Figure 2A** and 2B. We compared DNA alteration, TMB, RNA of immune-related molecules, PD-L1 expression, and immune cell score in the TME between these two groups. The most significant differences were the checkpoint molecule expression and immune-related pathway enrichment. Immune checkpoint molecules are referred as a series of gene that can help tumor cells escape immune surveillance. The well-known pathways are the interaction between CTLA-4 and CD80/86 (33) and the binding of PD-1 to PD-L1 to tumor cells (34). Other promising immune checkpoints mainly include B7 family inhibitory ligands as B7-H3(CD276), B7-H4(VCTN1), LAG3, TIGIT, and IDO1 (35). Although immune checkpoint molecules as predictors of responding to immunotherapy had been verified in other malignances (36–38), little is known about their roles in BTC. Our work, by preforming a 289 immune-related gene panel NanoString assay, has shown statistically increased *PD-1*, *PD-L1*, *LAG3*, *TIGHT*, and *IDO1* expression in responders even in this very small group of patients, indicating that patients harboring a highly immune tolerance tumor microenvironment may benefit from the immunochemical therapy. Other upregulation genes in responders were molecules including *PSMB9*, *CCL4*, *CCL5*, *CXCL10*, *CXCL11*, *CXCL13*, *IFI35*, *IFIT3*, and *IRF1* highly associated with antigen processing and presentation, T-cell migration, and IFN-gamma pathway. These data indicated a more diversity factors that might predict the prognosis of immune-combined therapy in ICC patients. We then generated a six-gene immune-related signature based on the most significantly changed genes/pathways between responders and non-responders. We found that this gene-set score was valuable for predicting the prognosis of ICC patients receiving immune-chemotherapy by a larger ICC cohort.

Interestingly, the predictors reported in previous research, such as single DNA alteration, DDR pathway-related gene mutation, and immune cells in TME, showed no significant difference between responders and non-responders in our study. DDR pathway-related gene mutation was only found positive in two responders. We considered that this difference may be due to the small cohort we tested or the diversity of different tumor types.

Peripheral blood lymphocytes have been reported as possible predictors of ICIs in several kinds of solid tumors, mainly in melanoma and NSCLC (39). In this study, we collected peripheral blood samples before and after the first cycle of immune-combined therapy in order to explore whether peripheral blood lymphocytes at baseline and after therapy were associated with efficiency of ICIs in ICC patients. However, in this study, we did not observe any association. Small sample size was the main reason. Peripheral blood lymphocytes are susceptible to infection and drug which could cause bias to the analysis.

TMB, defined as the number of somatic mutations per megabase of interrogated genomic sequence, is believed to be a key driver in the generation of immunogenic neopeptides displayed on major histocompatibility complexes (MHC) on the tumor cell surface that influence patient response to ICIs (40). Although the U.S. Food and Drug Administration (FDA) has approved pembrolizumab, an ICI targeting PD1, for individuals with TMB-High (defined as ≥10 mutations/Mb) solid tumors (41), evidence showed high TMB failed to predict response to ICIs across all cancer types including breast cancer, prostate cancer, and glioma (42). Recent research indicated that high levels of M1 macrophages and low resting dendritic cells in the TME characterized cancer types with high TMB power including cholangiocarcinoma (43). In our study, the result that two patients with high TMB displayed completely different responses to immune-combined therapy might support the possible fact that TMB was still not a determined predictor of immunotherapy in ICC.

PD-L1 expression on tumor cells has been reported highly heterogeneous due to different antibodies assayed, ranging from 4% to 100% (44). Our result showed an obviously high expression rate up to 91.6% (11/12) if the cutoff value of positive staining was set as >1%. We have found that PDL1 higher expression in pretreated tumor tissues did not achieve a better clinical outcome in ICC patients. In consistency with our result, PD-L1 expression at baseline was not associated with therapeutic efficacy in another phase 2 study with durvalumab (D) ± tremelimumab (T) and GemCis in chemo-naïve advanced BTC. However, an increased PD-L1 expression after one cycle of chemotherapy trended with improved PFS in their study, suggesting a therapeutic-responded PDL1 expression, rather than the baseline PDL1 levels, was represent for better prognosis (14). We did not conduct re-biopsy after therapy in patients due to the ethic restriction, but our *in vitro* experiment verified the induction of PDL1 expression in response to GEM/Oxp in ICC cells.

In summary, our work revealed that a group of molecules and immune-related pathways had a significant association with the efficacy of PD-1 antibody-combined GemCis in ICC patients. The six immune-related gene signatures, although generated based on a small cohort, displayed a good discriminatory ability of ICC patients receiving immunochemical therapy. We hope our work will contribute to other deepen studies of the therapeutics of BTC.

## Data availability statement

According to national legislation/guidelines, specifically the Administrative Regulations of the People’s Republic of China on Human Genetic Resources (http://www.gov.cn/zhengce/content/2019-06/10/content_5398829.htm, http://english.www.gov.cn/policies/latest_releases/2019/06/10/content_281476708945462.htm), no additional raw data is available at this time. Data of this project can be accessed after an approval application to the National Genomics Data Center ( NGDC, http://ngdc.cncb.ac.cn/databases), Please refer to http://ngdc.cncb.ac.cn/databases for detailed application guidance. The accession number was OMIX 001774.

## Ethics statement

This study was reviewed and approved by Eastern Hepatobiliary Surgery Hospital (EHBHK2020-01-008). The patients/participants provided their written informed consent to participate in this study.

## Author contributions

YG designed the study and obtained funding. TZ and YP wrote the manuscript. TZ revised the manuscript. YP performed cell-based experiment and helped to analyze data. TZ and ZY provided clinical tissue samples and analyzed the clinical data. DC and YS helped analyzing gene sequence data and all authors reviewed and approved the final article. All authors contributed to the article and approved the submitted version.

## Funding

This work was supported by the National Natural Science Foundation of China (81972290, 82073228), the Top-level Clinical Discipline Project of Shanghai Pudong (PWYgf2021-07) and Natural Science Foundation of Shanghai (20ZR1470000).

## Conflict of interest

Authors YS and DC were employed by Jiangsu Simcere Diagnostics Co., Ltd.

The remaining authors declare that the research was conducted in the absence of any commercial or financial relationships that could be construed as a potential conflict of interest.

## Publisher’s note

All claims expressed in this article are solely those of the authors and do not necessarily represent those of their affiliated organizations, or those of the publisher, the editors and the reviewers. Any product that may be evaluated in this article, or claim that may be made by its manufacturer, is not guaranteed or endorsed by the publisher.

## Supplementary material

Supplementary Figure 1 Increasing expression of PD-L1 and enrichment of inflammatory response in GEM-resistant TFK1 cells. **(A)** The cell viability of gemcitabine-resistant TFK1 and its parental cells in response to various dosages of GEM. **(B)** The EC50 were determined in response to various dosages of GEM. **(C)** The volcano plot of different genes between gemcitabine-resistant TFK1 and its parental cells. **(D)** The mRNA levels of *CD274* (PD-L1) in gemcitabine-resistant TFK1 group and its parentalgroup. **(E)** The protein levels of CD274 (PD-L1) in gemcitabine-resistant TFK1 group and its parentalgroup. **(F)** GSEA assay on differentiated pathways of gemcitabine-resistant TFK1 and its parental cells.

## Abbreviations

FGFR, Fibroblast growth factor receptors; TP53, Tumor Protein P53; KRAS, Kirsten rat sarcoma viral oncogene; IDH1, Isocitric Dehydrogenase1; PTEN, Gene of phosphate and tension homology deleted on chromosome ten; PI3KCA, Phosphatidylinositol 3-kinases; BAP1, BRCA1 associated protein 1; CDKN2A, Cyclin dependent kinase inhibitor 2A; SMAD4, SMAD Family Member 4; POLE, Polymerase epsilon; LAG3, Lymphocyte activation gene-3; TIGIT T, cell immunoreceptor with Ig and ITIM domains; IDO1, Indoleamine 2,3-dioxygenase 1; PSMB9 Proteasome 20S Subunit Beta 9.
