# Supplementary figures and images for: Immune-related RNA signature predicts outcome of PD-1 inhibitor-combined GEMCIS therapy in advanced intrahepatic cholangiocarcinoma
Source: Front Immunol. 2022 Sep 9;13:943066. doi: 10.3389/fimmu.2022.943066 (PMC9501891; doi:10.3389/fimmu.2022.943066)

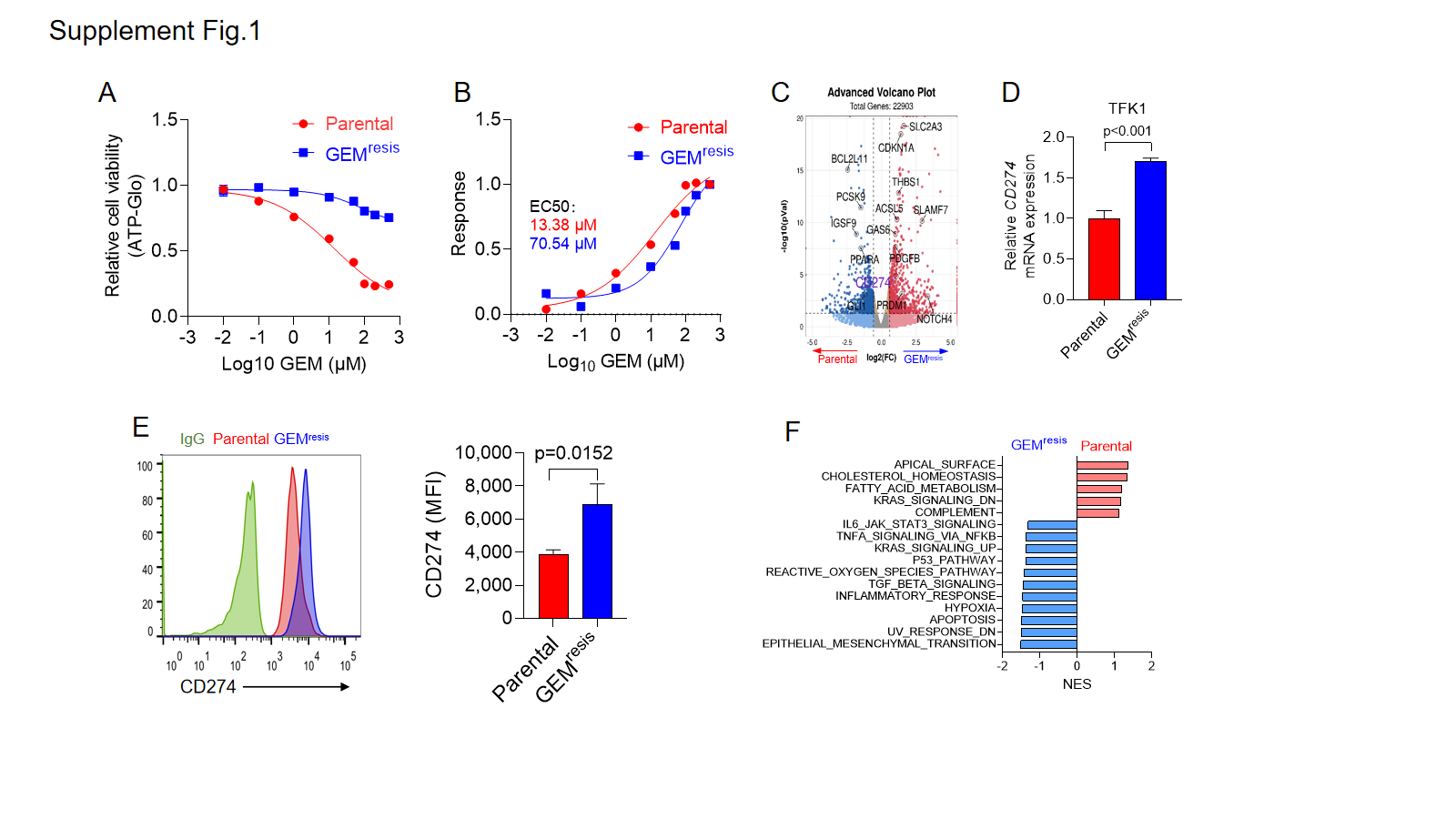

Supplement: Supplementary Figure 1 — Increasing expression of PD-L1 and enrichment of inflammatory response in GEM-resistant TFK1 cells. (A) The cell viability of gemcitabine-resistant TFK1 and its parental cells in response to various dosages of GEM. (B) The EC50 were determined in response to various dosages of GEM. (C) The volcano plot of different genes between gemcitabine-resistant TFK1 and its parental cells. (D) The mRNA levels of CD274 (PD-L1) in gemcitabine-resistant TFK1 group and its parentalgroup. (E) The protein levels of CD274 (PD-L1) in gemcitabine-resistant TFK1 group and its parentalgroup. (F) GSEA assay on differentiated pathways of gemcitabine-resistant TFK1 and its parental cells. [file Image_1.tif]
