# Supplementary material for: Immune-related RNA signature predicts outcome of PD-1 inhibitor-combined GEMCIS therapy in advanced intrahepatic cholangiocarcinoma
Source: Front Immunol. 2022 Sep 9;13:943066. doi: 10.3389/fimmu.2022.943066 (PMC9501891; doi:10.3389/fimmu.2022.943066)
Supplement: Supplementary file 4 [file Table_2.docx]

**Table s2 Q-PCR primers list**

| Name | Sequence |
| --- | --- |
| Human CD274-F | TGCCGACTACAAGCGAATTACTG |
| Human CD274-R | CTGCTTGTCCAGATGACTTCGG |
| Human HLA-A-F | AGATACACCTGCCATGTGCAGC |
| Human HLA-A-R | GATCACAGCTCCAAGGAGAACC |
| Human CXCL10-F | GGTGAGAAGAGATGTCTGAATCC |
| Human CXCL10-R | GTCCATCCTTGGAAGCACTGCA |
| Human CXCL11-F | AAGGACAACGATGCCTAAATCCC |
| Human CXCL11-R | CAGATGCCCTTTTCCAGGACTTC |
| Human TNFA-F | CTCTTCTGCCTGCTGCACTTTG |
| Human TNFA-R | ATGGGCTACAGGCTTGTCACTC |
| Human IL1b-F | CCACAGACCTTCCAGGAGAATG |
| Human IL1b-R | GTGCAGTTCAGTGATCGTACAGG |
| Human IFNA1-F | AGAAGGCTCCAGCCATCTCTGT |
| Human IFNA1-R | TGCTGGTAGAGTTCGGTGCAGA |
| Human ACTB-F | CACCATTGGCAATGAGCGGTTC |
| Human ACTB-R | AGGTCTTTGCGGATGTCCACGT |
