# Supplementary material for: Immune-related RNA signature predicts outcome of PD-1 inhibitor-combined GEMCIS therapy in advanced intrahepatic cholangiocarcinoma
Source: Front Immunol. 2022 Sep 9;13:943066. doi: 10.3389/fimmu.2022.943066 (PMC9501891; doi:10.3389/fimmu.2022.943066)
Supplement: Supplementary file 6 [file Table_4.docx]

**Table s4 Enrichment gene set and gene list**

| **Gene set** | **Gene list** |
| --- | --- |
| Response to biotic stimulus | **PSMB9, LAG3, CCL5, IFI35, SH2D1A, PSMB10, CD274,** HAVCR2, HLA-E, KLRK1, TLR8, HLA-DRB1, ARG1, IL17A, PVR, CD209, NECTIN2, KLRD1 |
| Regulation of innate immune response | **PSMB9, IRF1, LAG3, CCL5, IFI35, SH2D1A, PSMB10, FCGR2B, STAT1,** HAVCR2, HLA-E, KLRK1, TLR8, ARG1, NCR1, PVR, IFNG, CD209, NECTIN2, PTPN11,KLRD1 |
| T cell migration | **CXCL10, ITGAL, CXCL13, CCL5, CXCL11, CCL21, CCL20,** CCL2, TNFRSF14, ICAM1, PIK3CD, CXCL12,CXCR3, ZAP70, CCR2 |

Bold represents core gene
